# Regulatory T cells in COVID-19

**DOI:** 10.14336/AD.2021.0709

**Published:** 2021-10-01

**Authors:** Huan Wang, Zhao Wang, Wen Cao, Qianqian Wu, Yujia Yuan, Xiangjian Zhang

**Affiliations:** ^1^Department of Neurology, Second Hospital of Hebei Medical University, Shijiazhuang, Hebei 050000, China; ^2^Hebei Collaborative Innovation Center for Cardio-cerebrovascular Disease, Shijiazhuang, Hebei 050000, China; ^3^Hebei Vascular Homeostasis Key Laboratory for Neurology, Shijiazhuang, Hebei 050000, China

**Keywords:** COVID-19, SARS-CoV-2, Regulatory T cells, inflammation, immune responses

## Abstract

The outbreak of coronavirus disease 2019 (COVID-19) is caused by the infection of severe acute respiratory syndrome coronavirus 2 (SARS-CoV-2), which leads to the disruption of immune system, exacerbated inflammation, and even multiple organ dysfunction syndrome. Regulatory T cells (Tregs) are an important subpopulation of T cells that exert immunosuppressive effects. Recent studies have demonstrated that the number of Tregs is significantly reduced in COVID-19 patients, and this reduction may affect COVID-19 patients on several aspects, such as weakening the effect of inflammatory inhibition, causing an imbalance in Treg/Th17 ratio, and increasing the risk of respiratory failure. Treg-targeted therapy may alleviate the symptoms and retard disease progression in COVID-19 patients. This study highlights the recent findings on the involvement of Tregs in the regulation of immune responses to COVID-19, and we hope to provide novel perspectives on the alternative immunotherapeutic strategies for this disease that is currently prevalent worldwide.

## 1.Introduction

The outbreak of severe acute respiratory syndrome coronavirus 2 (SARS-CoV-2) infection, which is highly contagious and pathogenic, has raised substantial concern [1]. The acute respiratory disease caused by SARS-CoV-2 is known as coronavirus disease 2019 (COVID-19), and it induces severe and even fatal respiratory syndromes that damage multiple organ systems in the body. A number of studies have revealed that the adaptive immune system of COVID-19 patients is defective (as manifested by lymphopenia), and the degree of lymphopenia correlates with the severity of SARS-CoV-2 infection [2,3,4]. Regulatory T cells (Tregs), an essential component of the adaptive immune system, may play an important role in the regulation of immune responses to COVID-19.

## 2. Biological characteristics of Tregs

As a subset of CD4^+^ T cells, Tregs are critically involved in the regulation of immune responses to diseases, such as negatively modulating the activation, proliferation, and effector functions of various immune cells to maintain self-tolerance and immune homeostasis [5]. Tregs can be divided into two categories based on their origin: thymus-derived Treg or naturally occurring Treg, whose differentiation depends on its high-affinity interaction with the autopeptide/MHC II complex in the thymus [6], and peripheral-derived Treg or induced Treg, which are induced by naive CD4+ T cells in the periphery or in vitro [7].

**Figure 1.**
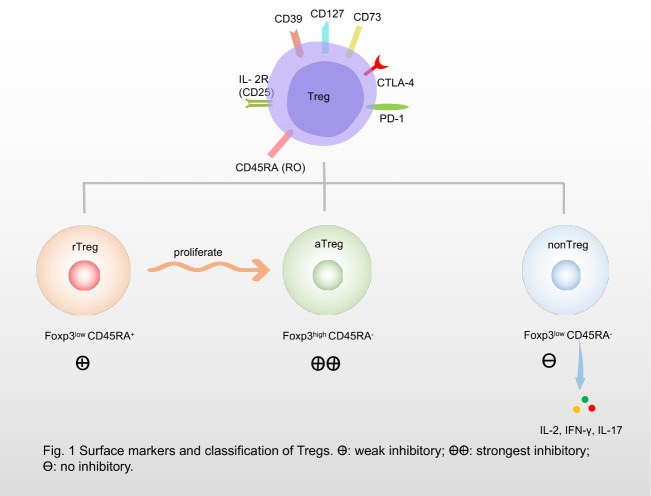
Surface markers and classification of regulatory T cells (Tregs). Tregs can express Forkheadbox P3 (Foxp3), CD25, Cytotoxic T lymphocyte associated antigen-4 (CTLA-4), CD127, programmed death 1 (PD-1), CD39, CD73, and some other costimulatory molecules. According to the expression of Foxp3 and CD45RA, Tregs can also be divided into three subgroups: resting Treg cells (rTreg, CD45RA^+^ Foxp3^low^), activated Treg cells (aTreg, CD45RA^-^ Foxp3^high^) and cytokine secretion non-suppressive T cells (nonTreg, CD45RA^-^ Foxp3^low^). The aTregs have the strongest inhibitory function; rTregs are naïve Tregs with weak inhibitory activity and can proliferate and differentiate into aTreg; nonTregs are capable of secreting a series of cytokines including interleukin (IL)-2, interferon-γ (IFN-γ), and IL-17, but have no inhibitory activity.

Tregs differ phenotypically according to their cell surface markers [8] (Fig. 1). Forkhead box P3 (Foxp3) is a transcriptional regulator that controls the development and function of Tregs [9] and is expressed by almost all inhibitory Tregs. Its expression level determines the immunosuppressive properties of Foxp3^+^ Treg [9]. Tregs also express CD45RO and CD45RA, which are mutually exclusive in Foxp3^+^ Tregs [5]. According to the expression levels of Foxp3 and CD45RA, Tregs can also be divided into three subpopulations: resting Tregs (rTregs, CD45RA^+^ Foxp3^low^), activated Tregs (aTregs, CD45RA^-^ Foxp3^high^), and cytokine secreting non-suppressive T cells (nonTregs, CD45RA^-^ Foxp3^low^). The aTregs have the strongest inhibitory potential. The rTregs are naïve Tregs with weak inhibitory activity, but they can proliferate and differentiate into aTregs in vivo or in vitro [10]. The nonTregs are capable of secreting a series of cytokines including interleukin (IL)-2, interferon-γ, and IL-17, but without inhibitory activity. CD45RO^+^ Foxp3^high^ Tregs, called effector Tregs, are a subset of activated and functionally differentiated Tregs that can be derived from CD45RA^+^ Foxp3^low^-naïve Tregs after antigen stimulation [5]. CD25 is a component of high-affinity IL-2 receptor (IL-2R) expressed in almost all types of Foxp3^+^ Tregs, hence, a low-dose IL-2 preferentially affects Tregs [11]. CD127, a receptor of IL-7, is expressed on the Treg surface and is negatively correlated with the expression of Foxp3 [12]. Furthermore, programmed death 1 (PD-1), CD39, CD73, cytotoxic T lymphocyte-associated antigen-4 (CTLA-4), and some other costimulatory molecules are also selectively expressed on the surface of Tregs.

Tregs play a critical role in maintaining immune homeostasis and limiting immunopathology. In general, Tregs are the first-line of defense against uncontrolled inflammation and viral infections [13], which suppress CD4^+^ and CD8^+^ T-cell responses and reduce the infiltration of NK cells, eosinophils, and neutrophils [14,15].

## 3.Changes in the expression levels of Tregs in COVID-19 patients

The alterations in Tregs have been widely observed in COVID-19 patients (Table 1). Chen et al. found that the expression levels of CD4^+^ CD25^+^ CD127^low^ Tregs and CD45RA^+^ Tregs were reduced in both moderate and severe COVID-19 patients. The proportion of CD45RA^+^ Tregs was significantly lower in patients with severe cases than in those with moderate cases (0.5% vs 1.1%), while the proportion of CD45RO^+^ Tregs were comparable between moderate and severe COVID-19 patients [2]. The loss of CD45RA^+^ Tregs and increase in the IL-10 levels may lead to hyperimmunity in severe COVID-19 patients, possibly associated with high mortality among these patients [16]. Another study revealed that Treg levels in COVID-19 patients, particularly in those with severe cases, were remarkably lower than normal [17]. A study including 19 children with COVID-19 showed that the expression levels of CD45RA^+^ Tregs were obviously suppressed during the acute phase (0-3 days after onset) and returned to normal during the recovery phase (17-24 days after onset) [18]. The excessive reduction of Treg levels tends to cause persistent and severe tissue damage.

**Table 1:** The studies of Tregs in COVID-19 patients.

Study	Country	number of cases	average age	The changes of Treg	Severity of illness	Proportion of COVID-19 patients with reduced Tregs	The extent of Tregs reduced/increased
Chen et al^[2]^	China	21	57	CD4^+^ CD25^+^ CD127 ^low^ Treg and CD45RA^+^ Treg reduce	severe:11 moderate:10	almost all moderate and severe COVID-19 patients	significantly lower in severe cases than in moderate cases
Wang et al^[16]^	China	65	57	CD45RA^+^ Treg reduce	mild:30 severe:20 extremely severe:15	Not mention	significantly decreased in severe patients, especially in those with extremely severe illness
				CD45RO^+^ Treg increase			Increase with the severity of the disease
Qin et al^[17]^	China	452	58	CD3^+^CD4^+^ CD25^+^CD127^low^ Treg reduce	severe:286 mild to moderate:166	Not mention	Not mention
Jia^[18]^	China	19	9	Tregs (% of CD4^+^ T cells) reduce	mild	Not mention	Not mention
Tan et al^[19]^	China	56	54	Tregs increase	severe:25 mild to moderate:31	Not mention	percentage of Tregs in lymphocytes increased significantly in both groups
Julika et al^[20]^	Belgium	49	61	IL-10-producing Treg increase	severe:20 mild to moderate:23 healthy:6	Not mention	5-fold increase in severe cases
Yang et al^[21]^	China	1	30	CD45RA^-^ FoxP3^high^ Treg increase	asymptomatic SARS-CoV-2 infected individual	Not mention	4.4-fold of that in healthy controls
Sara et al^[22]^	Italy	39	64	higher percentage of CD45RA^+^CCR7^+^ Treg	Not mention	Not mention	Not mention
				Activated CD45RA^-^CCR7^+^ Treg increase	Not mention	Not mention	Not mention

However, opposite findings have also been reported regarding the changes in Treg levels in COVID-19 patients. Tan et al. reported that Treg levels were remarkably increased in patients with mild cases and moderately increased in those with severe cases, indicating immunosuppression in COVID-19 patients. The expression of CD25 on the Treg surface was enhanced in moderate and severe COVID-19 patients, especially in those with severe cases, indicating the elevated activity and function of Tregs [19]. Julika et al. also found that Treg levels were increased in moderate and severe COVID-19 patients, and the level of IL-10-producing Tregs was particularly high in severe COVID-19 patients. The production of IL-10 may be a hallmark of Tregs activation in certain tissues such as the lungs, which can reflect the severity of the disease [20]. Yang et al. reported an asymptomatic patient with SARS-CoV-2 infection [21] with CD3^+^CD8^-^CD4^+^CD127^-^CD25^+^ Treg surface phenotypes. The levels of aTregs were 4.4 times higher than that of healthy controls at day 7, peaked at day 22, and decreased at day 28. The high proportion of incompetent T cells during the early phase of asymptomatic infection suggested that aTregs may inhibit the activation and function of T cells during the initiation phase of COVID-19 [21]. Moreover, some COVID-19 patients showed a reduction in the levels of CD45RA^+^CCR7^+^ Tregs and an increase in the levels of activated CD45RA^-^CCR7^+^ Tregs, in the absence of Foxp3 [22].

## 4.Possible causes of Treg changes in COVID-19

Under normal conditions, Tregs migrate into inflamed tissues to dampen inflammatory responses and accelerate tissue repair [23]. COVID-19 patients with acute respiratory distress syndrome (ARDS) have protracted hospital stays owing to excessive systemic inflammation (cytokine storm) and delayed pulmonary repair, which is partly due to the decreased expression of Tregs or defects in these cells [24]. Recent studies unveiled a significant increase in the levels of Tregs in lymphocytes, indicating a positive anti-inflammatory response. Besides, Sarah et al. concluded that SARS-CoV-2 caused an impairment in the transport of Tregs from the circulation to the respiratory tract, leading to lung injury as well as accumulation of Tregs in the circulation. Hence, the immunotherapy that promotes the entry of circulating Tregs into the respiratory tract may reduce the risk of developing COVID-19 [25]. The reduction of circulating Tregs in COVID-19 patients may be caused by the following factors.

First, the negative regulatory mechanism mediated by Tregs is activated after SARS-CoV-2 infection. However, in critically ill patients, the consistent and overwhelming inflammatory responses eventually leads to lymphocyte apoptosis and even to lymphocyte failure in the late stage of infection [19]. Meanwhile, extreme inflammatory conditions may cause Treg destabilization, depriving them of the ability to express Foxp3 and immunosuppressive functions and transforming them into effector T cells [26]. Therefore, the alterations of Tregs may be related to the severity and stage of the disease.

Second, SARS-CoV-2 invasion induces significantly increased level of serum IL-6, which promotes the differentiation of naïve CD4^+^ T cells into Th17 cells as well as inhibits the expression of Tregs, resulting in an imbalance of the Treg/Th17 ratio [27,28]. In addition, some COVID-19 patients develop other complications such as diabetes, which cause CD4^+^ T cells to differentiate into Th1 and Th17 rather than into Tregs, leading to diminished immunosuppression [26].

Third, SARS-CoV-2 can induce multiple activation and differentiation of CD4+ T cells, during which the decrease in the levels of Foxp3 accelerates T-cell activation and death [29]. The invasion of SARS-CoV-2 is dependent on the presence of spike (S) protein on the surface of the virus [30]. The pro-protein convertase furin activates the S-protein, and its T-cell-specific deletion effect results in the impairment of FoxP3 and TBX21 that promote Treg differentiation [31]. In severe COVID-19 patients, CD4^+^ T cells are hyperactivated, but Foxp3 expression is repressed. Significant proportions of T cells are activated, proliferate, and undergo rapid death before differentiating into Tregs [29].

Forth, the gene sequence of SARS-CoV-2 is highly homologous to that of SARS-CoV, which has the capability to infect T lymphocytes [32]. It is reasonable to assume that SARS-CoV-2 may also directly attack the lymphocytes, causing these cells to die, subsequent lymphopenia, and weakened immune response [33].

Last, hypoxia in the lungs of severe COVID-19 patients can activate hypoxia-inducible factor-1α, which binds to Foxp3 and targets the proteasome for degradation [34], leading to the abrogation of Foxp3 autoregulatory transcriptional loop and thereby impeding the differentiation of Tregs [35].

Tregs are involved in the regulation of immune responses to COVID-19. Therefore, it is important to understand the impacts of Tregs on COVID-19 patients and to develop new therapeutic approaches targeting Tregs.

## 5.Potential impacts of Tregs in COVID-19

**Figure 2.**
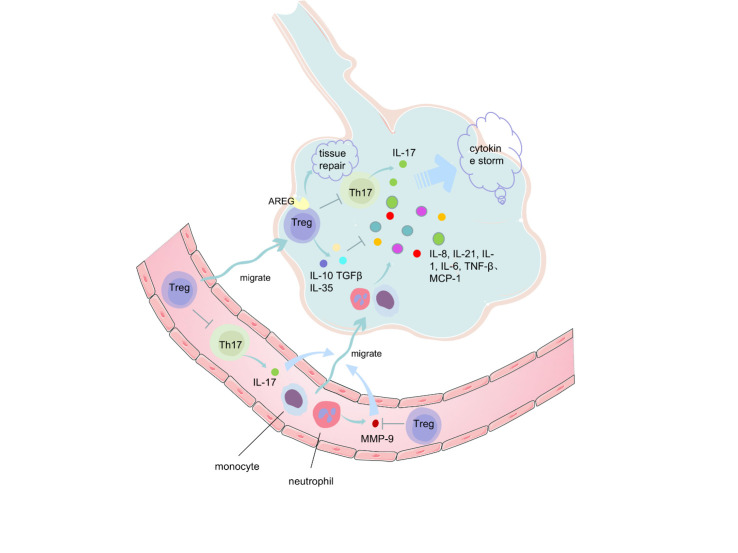
Regulatory T cells (Tregs) may play a role in COVID-19 in several ways. (1) Tregs promote lung tissue repair by expressing amphiregulin (AREG). (2) Circulating Treg may migrate to the lung, inhibit tumor necrosis factor (TNF)-α, interleukin (IL)-6 and other cytokines by producing IL-10 and transforming growth factor (TGF)-β. Besides, Tregs inhibit Th17 cells, IL-17 recruit neutrophils and monocytes to the site of infection and activate downstream factors such as IL-8, IL-21, IL-1, IL-6, TNF-β?monocyte chemotactic protein (MCP)-1, followed by the uncontrolled systemic inflammation (cytokine storm). (3) Tregs could inhibit the migration of inflammatory cells to lung tissue by inhibiting neutrophil-derived metalloproteinases (MMP)-9.

Tregs may affect the immune response after COVID-19 in many ways (Fig. 2).

### 5.1 Tregs reducing the susceptibility to SARS-CoV-2

Studies have shown that older men infected with SARS-CoV-2 have a relatively higher mortality rate. Older diabetic patients are more susceptible to SARS-CoV-2 infection and have a much higher mortality rate than average, which is associated with the decreased Treg levels [36,37]. Güneş et al. noted that the low prevalence of COVID-19 among children and young adults may be associated with the high activity of their Tregs [38]. X chromosomes can encode immune regulatory genes such as Foxp3; Pcnti et al. suggested that women are less susceptible to SARS-CoV-2 infection than men, probably because women have two X chromosomes, resulting in a lower viral load in women than in men [39]. In the mouse models of viral pneumonia, aging can lead to the loss of pro-repair transcriptional programs in Tregs, causing age-related maladaptive T-cell responses, such as effector T-cell differentiation, cell cycle arrest, and DNA damage responses. Aging can also result in the upregulation of the inflammatory responses mediated by Th1 and Th17 cells as well as lung inflammation and injury [40].

### 5.2 Tregs promoting lung tissue repair after SARS-CoV-2 infection

A previous study demonstrated that Tregs have the capacity to directly exert tissue repair function and preserve the lung structural and functional integrity during the early stages of influenza infection by producing amphiregulin (AREG), which is induced by IL-18 and IL-33. Selective Treg deficiency in AREG leads to severe acute lung injury and decreased blood oxygen concentration during the period of influenza virus infection. The selective loss of AREG can cause severe lung damage and decreased lung function [41]. The tissue repair ability of Tregs does not depend on their immunosuppressive function and anti-inflammatory function.

In a prospective cohort of COVID-19 patients, the expression of Notch-4 in circulating Tregs increased, which suppressed the release of IL-18 induced AREG, thus restraining AREG-dependent tissue repair and promoting severe lung injury [42]. This finding suggests that Tregs may be a potential therapeutic target for COVID-19.

### 5.3 Imbalance in Treg/Th17 ratio aggravating the cytokine storm associated with COVID-19

Decrease in the levels of peripheral Tregs in COVID-19 patients can disrupt the balance between regulatory and effector arms of the immune system. Reduction in the Treg/Th17 ratio is a pro-inflammatory and risk factor of ARDS and serves as a prognostic marker for ARDS [43]. Severe COVID-19 patients exhibit a decrease in the levels of Tregs and an increase in the levels of Th17 cells [16,17,22], the imbalance in the Treg/Th17 cell ratio plays an important role in the uncontrolled systemic inflammation (cytokine storm) in these patients [27]. In pregnant women with COVID-19, the imbalance in Tregs/Th17 cell ratio and the subsequent systemic inflammation may contribute to the pathogenesis of pregnancy complications, such as pregnancy loss, preterm delivery, and preeclampsia (PE) [31], because the balance in the ratio between these cells is essential for maintaining a healthy fetal implantation and pregnancy development [44]. The decrease in Treg levels and the relative increase in Th17 cell levels can amplify the inflammatory response. Th17 cells recruit neutrophils to the infection site and activates downstream cytokines and chemokines, including IL-8, IL-21, IL-1, IL-6, TNF-β, and MCP-1 [45], subsequently recruiting more effector cells and releasing massive inflammatory cytokines that amplify the cytokine storm [27].

Tregs may play a key role in preventing the cytokine storm associated with severe respiratory disease caused by viral infection [46]. In viral pneumonia, Tregs can inhibit the activity of NK cells, macrophages, and CD8^+^ cytotoxic T cells by secreting IL-10, TGF-β, and IL-35, thereby reducing the severity of cytokine storm. IL-10 is crucial in combating excessive inflammatory response [47]. Circulating Tregs can inhibit the release of TNF-α, IL-6, and other cytokines through the production of IL-10 and TGF-β. In addition, Tregs inhibit the production of Th17 cells in order to alleviate cytokine storm-induced lung injury [48], indicating that Tregs may have a therapeutic potential against COVID-19.

### 5.4 Tregs improving respiratory failure by inhibiting MMP-9

Respiratory failure due to ARDS is an important cause of high mortality in COVID-19 patients. It was recently found that serum matrix metalloproteinases-9 (MMP-9) level was significantly higher at admission and 3-5 days after onset in patients with COVID-19. Therefore, MMP-9 can be used as an early indicator of respiratory failure in COVID-19 patients [49]. Neutrophils are a major cellular source of MMP-9 [49], and the autopsy of deceased COVID-19 patients showed marked neutrophil infiltration in the pulmonary capillaries [50]. Neutrophil-derived MMP-9 may aggravate inflammation and the degradation of alveolar capillary barrier, further stimulating the migration of inflammatory cells to the lung tissue and causing more severe injury [51]. Tregs specifically inhibits the MMP-9 production by neutrophils [52]. The reduction of Treg levels in COVID-19 patients attenuates their inhibitory effect on MMP-9, thus exacerbating lung injury. Therefore, Tregs may be a research target for the prevention of respiratory failure in COVID-19 patients.

### 5.5 Tregs inhibiting the overactive immune system of COVID-19 patients

In severe COVID-19 patients, T cells are highly activated but Foxp3 expression is inhibited, while hyperactivated CD25^+^ T cells proliferate and die rapidly before differentiating into Tregs [29]. The reduction of Treg levels may contribute to the overactive immune system and lung injury in patients with COVID-19 [53]. However, this frequent renewal of hyperactivated CD25^+^ T cells may lead to immunothrombosis, another feature of severe COVID-19 [54].

### 5.6 Neuropilin-1 receptor expressed by Tregs inducing anosmia

Two teams have independently discovered an alternative pathway for SARS-CoV-2 entry mediated by neuropilin-1 receptor (NRP1), which also binds to the S protein in the virus [55,56]. NRP1 is expressed on the surface of Tregs. Although NRP1 can mediate SARS-CoV-2-induced anosmia, Tregs may better suppress the cytokine storm in COVID-19 patients [57].

## 6. Potential of Treg-based therapies in the management of COVID-19 patients

Most current clinical trials on the treatment of COVID-19 patients involve antiviral drugs (such as ribavirin, favipiravir, and remdesivir), immunologic agents (such as tocilizumab and sarilumab), and plasma from recovered patients, but no specific therapeutic agent has been developed yet. Treg-related therapies have already been established in a variety of inflammatory disease models and autoimmune diseases [58], and excessive inflammation is the primary cause of disease severity and death in COVID-19 patients. Therefore, Treg-based immunotherapy may also provide feasible regimens for treating COVID-19.

The adoptive transfer of Tregs has been demonstrated to suppress fibroproliferation and improve outcome in lung injury animals [59, 60], suggesting that increasing the levels of Tregs in patients with ARDS would be an ideal strategy to treat the advanced stages of COVID-19. In ARDS, the downregulation of Akt in T cells promotes their differentiation into Tregs, thereby limiting inflammation and scar formation and promoting angiogenesis [61]. The inhibition of Akt pathway is expected to increase the Treg levels in order to suppress pathological inflammation, cytokine storm, fibrosis, and platelet activation associated with COVID-19 [62]. Recently, the physicians from Johns Hopkins University reported that two COVID-19 patients with ARDS were treated two or three times with allogeneic, off-the-shelf, cord blood-derived, ex vivo expanded Tregs, which significantly downregulated the inflammatory mediators and achieved promising outcomes. Further clinical trials on the use of cord blood-derived Tregs for ARDS-associated-COVID-19 will be conducted [63].

The proliferation and inhibitory functions of Tregs depend on the level of IL-2, which stabilizes the expression of Foxp3 and regulates the production of surface molecules such as CTLA-4 and tumor necrosis factor receptor. Treatment with low-dose IL-2 may control SARS-CoV-2-related ARDS by inducing the proliferation of Tregs. The ongoing clinical trials (ClinicalTrials.gov Identifier: NCT04357444) will explore the efficacy of low-dose IL-2 in SARS-CoV-2-related ARDS, in the hope of providing clinically feasible treatment for SARS-CoV-2-related ARDS.

A prospective cohort study demonstrated that expression of Notch-4 is elevated in the circulating Tregs of COVID-19 patients, which can be used as an indicator of disease severity and may be associated with high mortality. Inhibition of Notch-4 signaling pathway can promote tissue repair and reduce inflammatory response in patients with Treg-mediated viral infectious pneumonia, which is closely related to increased production of AREG by Tregs [42]. This finding suggests that the intervention along the Notch4-AREG axis may be a feasible treatment strategy for COVID-19.

## 7. Conclusion

To date, the global outbreak of COVID-19 has not been effectively controlled, and a variety of vaccines are under development. Hence, vigorous measures are urgently needed to prevent infection, avoid aggravation of the disease, and lower the risk of death. As a type of immunomodulatory cells, Tregs have emerged as the target in a series of therapies for autoimmune and inflammatory diseases. According to the changing characteristics of Tregs and their potential impacts on COVID-19 patients, Tregs are expected to become a novel target for COVID-19 treatment, which should be carefully evaluated in further studies and clinical trials.
